# P-189. Clinical Presentations, Treatments, Outcomes, and Epidemiology of Neurocysticercosis: A 30 Year Pilot Study of Newly Diagnosed Hospitalized Patients in Florida

**DOI:** 10.1093/ofid/ofae631.393

**Published:** 2025-01-29

**Authors:** Maggie Zawoy, Waverly Leonard, Norman Beatty, Elise M O’Connell

**Affiliations:** University of Florida College of Medicine, Gainesville, Florida; University of Florida College of Medicine, Gainesville, Florida; University of Florida, Gainesville, Florida; National Institute of Allergy and Infectious Diseases, Bethesda, Maryland

## Abstract

**Background:**

Neurocysticercosis (NCC) is a parasitic disease of the CNS caused by the pork tapeworm, *Taenia solium*, and is the leading cause of acquired seizure disorder worldwide. This study is the first to explore the clinical characteristics and courses of NCC patients in Florida.

**Figure 1:**
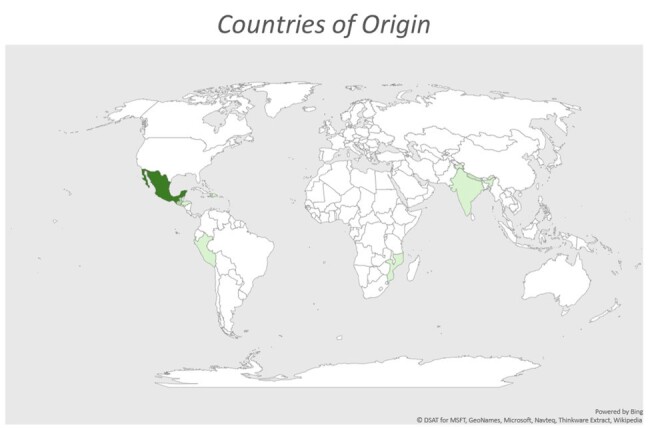
Countries of Origin 76% of patients reported a country of origin outside of the United States (Mexico, 12; Guatemala, 4; Haiti, Nepal, El Salvador, 2; Dominican Republic, Honduras, India, Mozambique, Nepal, Peru, 1) The remainder of patients did not specify a country of origin.

**Methods:**

The inclusion criteria for this retrospective chart review included any patient presenting to UF Health Shands Hospital, Gainesville, FL, with symptomatic NCC diagnosed between June 1993-June 2023 given an ICD-9 or ICD-10 code of NCC confirmed by imaging, serology, or biopsy.

**Table 1:**
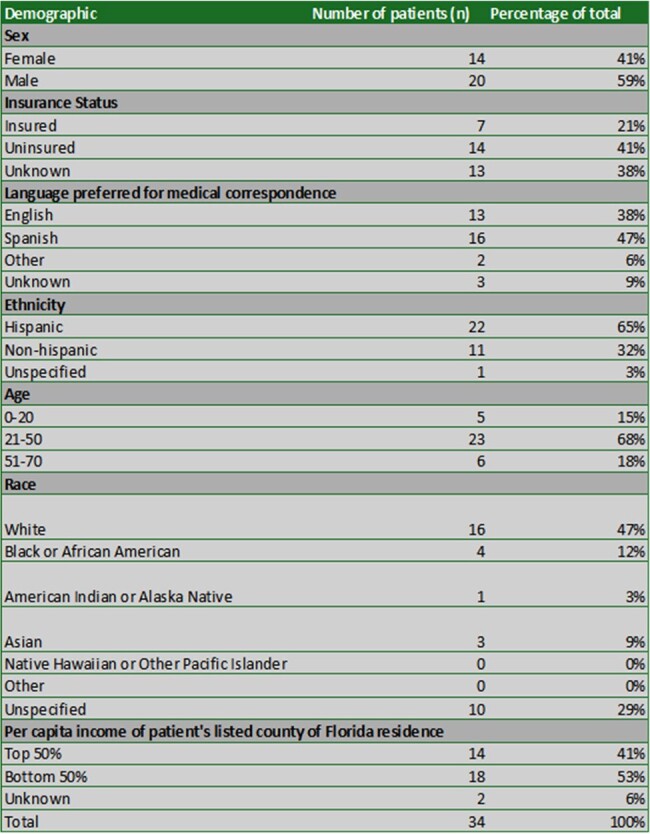
Epidemiological Characteristics of NCC Patients Selected epidemiological characteristics of 34 identified patients represented as percentages.

**Results:**

34 patients met inclusion criteria. Demographic characteristics of these patients included country of origin (76% non-USA, 24% unspecified), sex (59% Male, 41% Female), ethnicity (65% Hispanic, 32% non-Hispanic, 3% unspecified), age (68% 21-50, 18% 51-70, 15% 0-20), race (47% white, 12% black/AA, 9% Asian, 3% AIAAN, 29% unspecified), insurance status (41% uninsured, 21% insured, 38% unspecified), language preferred for medical correspondence (47% Spanish, 38% English, 6% other, 9% unspecified), and per capita income of Florida county of origin (53% bottom 50%, 41% top 50%, 6% unspecified).

Select pre-, intra-, post-hospitalization features and outcomes were recorded. 52% of patients' cysts were staged. Symptoms at time of presentation included headaches (25%), seizures (20%), systemic symptoms (e.g. fever, chills, malaise) (15%), and CNS deficits (10%). 97% of patients reported NCC symptoms prior to receiving an NCC diagnosis and 72% of patients presented to at least one healthcare facility for similar symptoms without receiving a diagnosis of NCC. Out of the 52% who were staged, 56% received incorrect medical management; these patients either received medications for inactive infection (18%) or did not receive proper medication for active infection (38%). 21% of patients were readmitted at least once for NCC symptoms or complications. 18% of patients stayed in the hospital for more than 10 days.

**Figure 2:**
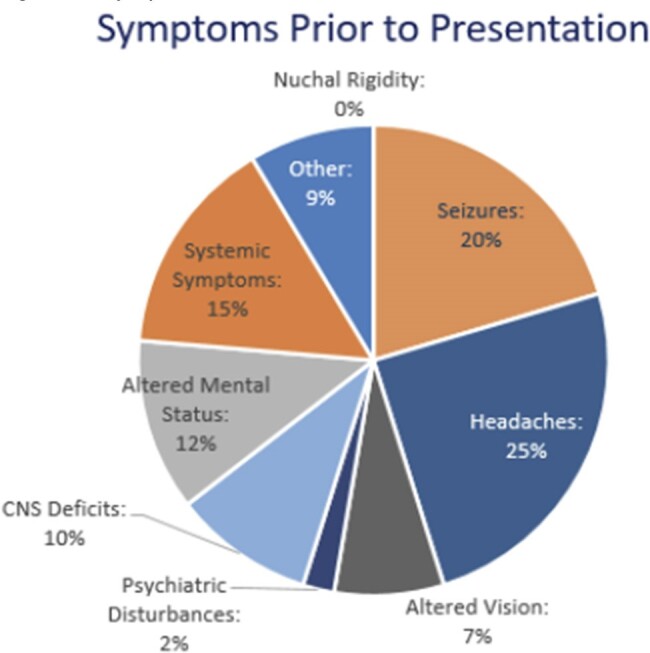
Symptoms Prior to Presentation Symptoms patients reported experiencing prior to the time of their diagnostic presentation.

**Conclusion:**

This research identifies a unique population of patients burdened by NCC and characterizes delays in patient presentation and diagnosis. This may aid clinicians in identifying common patient presentations associated with NCC to promote prompt and accurate diagnosis, appropriate treatment, and close follow-up.

**Table 2:**
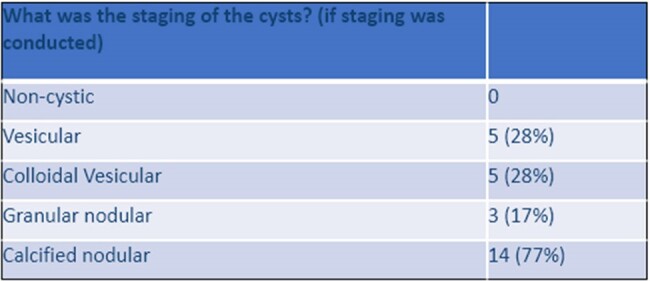
NCC Cyst Staging Cyst staging was performed for 53% of patients (18 in total); 27 cysts in total were staged representing multiple patients with more than one visible NCC cyst that was staged.

**Disclosures:**

**All Authors**: No reported disclosures

